# Evaluation of kidney injury molecule-1 as a disease progression biomarker in diabetic nephropathy

**DOI:** 10.12669/pjms.35.4.154

**Published:** 2019

**Authors:** Fatima Abid Khan, Syeda Sadia Fatima, Ghulam Mustafa Khan, Sana Shahid

**Affiliations:** 1 Fatima Abid Khan, Department of Physiology, Jinnah Sindh Medical University, Karachi Pakistan; 2 Syeda Sadia Fatima, Department of Biological and Biomedical Sciences, Aga Khan University, Karachi, Pakistan; 3 Ghulam Mustafa Khan, Department of Physiology, Basic Medical Sciences Institute, Jinnah Post Graduate Medical Centre, Karachi, Pakistan; 4 Sana Shahid, Department of Physiology, Sir Syed College of Medicine for Girls, Karachi, Pakistan

**Keywords:** Diabetes Mellitus, Diabetic Nephropathy, KIM-1

## Abstract

**Background & Objective:**

Kidney Injury Molecule-1 (KIM-1) is a peptide whose release into circulation is specific to tubular injury. This study aimed to estimate levels of kidney injury molecule-1 in diabetic patients with and without kidney disease. And evaluate the role of KIM-1 as an early screening marker of progressive kidney injury.

**Methods:**

This follow-up study included n=85 subjects from the diabetic clinic of Jinnah Post Graduate Medical Center (JPMC) in collaboration with Aga Khan University from November 2016 till September 2017. They were divided as: i) Group A1 (n=30) participants with diabetes for <5 years without microalbuminuria ii) Group A2 (n= 30) subjects with diabetes for 6-10 years with microalbuminuria; iii) Group B (n=25) subjects as healthy control group. All study participants were followed for 6 months and their blood glucose, urea, creatinine, electrolytes, albuminuria and serum KIM-1 were assayed.

**Results:**

High KIM-1 at baseline was present in group A2 patients as compared to controls and group A1 (p<0.001). Higher levels were seen after six months in group A1 along with the presence of micro albuminuria (p<0.001) suggesting kidney damage. Moderate positive association were seen for KIM1 with creatinine levels (r=0.530; p<0.001), and HbA1c (r=0.576; p<0.001) in all patients. While a strong positive association was seen for blood urea nitrogen as a marker for kidney function both at baseline (r= 0.728; p=0.000) and follow up (r=0.747; p=0.001). Multiple logistic regression controlling for age showed that KIM1 was independently associated with BUN (r=0.727; p<0.001), creatinine (r=0.510; p<0.001) and HbA1c (r=0.401; p=0.008) in all groups.

**Conclusion:**

Rising KIM-1 levels with progressive kidney damage with or without derangement of kidney function is reported in this study. This finding may pave a way towards identifying KIM1 as a prognostic marker for kidney injury.

## INTRODUCTION

Diabetic nephropathy (DN) is a disease of multifactorial pathogenesis involving several pathways which ultimately progress to reduction of renal functions.^1^,^2^ Estimated figures show that around 10-30% of type I diabetes mellitus and 15-40% of type II diabetes mellitus patients suffer from DN.^2^ Activation of protein kinase C, increased production of advanced glycosylation end products (AGEs), and diacylglycerol synthesis are proposed as some of the triggers for progression of DN. Other mechanism of renal damage in diabetes includes hemodynamic alterations such as glomerular hyper filtration, micro albuminuria and the metabolic changes.^3^,^4^

Diagnosis and prognosis of DN largely depends on traditional markers such as estimated glomerular filtration rate (e-GFR) and albuminuria however, the gold standard for diagnosing DN is performing a renal biopsy which is less commonly used due to its invasive nature. Contrarily, micro albuminuria has been used as a diagnostic marker for >30 years for renal injury. But recent data suggests the inability of micro albuminuria changes to foresee the nephropathy progression because in at least 33% of patients renal decline has already ensued.^2^,^4^ Similarly, derangement of GFR is an indicator of DN but it can only detect the disease when it has progressed to incurable stage.^5^,^6^ Apart from these; conventional methods such as assessment of blood urea nitrogen and creatinine are routinely used but are quite insensitive and nonspecific, especially in the setting of acute kidney injury (AKI) in perspective of DN. This raises the urgent need of new and novel biomarkers which can precisely predict AKI.^5^,^6^

Kidney injury molecule-1 (KIM-1) is a type I trans membrane glycoprotein (having a six-cysteine immunoglobulin- like domain two N -glycosylation sites)^7^, that is expressed on renal proximal tubule epithelial cells and is believed to play a role in renal tubulo-interstitial damage. The normal reference level of serum KIM-1 reported is as <1 ng/ml.^8^ Based on the observations and current knowledge it can be asserted that KIM-1 plays significant role in renal regeneration processes. This is because KIM-1 being a differentiation and proliferation marker is totally undetected in normally functioning renal system and only expressed in tubular cells after renal injury.^9^,^10^ Post ischemia/injury removal of debris (apoptotic and necrotic cells), from the luminary surfaces is mandatory for remodeling, restoration processing and integrity of renal tissues. Ichimura et al. in 1998 postulated that the mechanism of removal of debris is dependent on the role of KIM-1.^11^ KIM-1-expressing epithelial cells exhibit strong phagocytic ability in vivo. Furthermore; it is KIM-1 that specifies the site of dead cells and simultaneously mediates these phagocytic properties.^11^ Therefore, the aim of this study was to estimate levels of kidney injury molecule-1 in diabetic patients with and without kidney disease. And evaluate the role of KIM-1 as an early screening marker of kidney injury.

## METHODS

This follow up study included 112 participants between the age of 35-65 year from the diabetic clinic of Jinnah Post Graduate Medical Center (JPMC) in collaboration with Aga Khan University from November 2016 till September 2017. Out of the n=112 patients, we were able to follow n=85 patients with a drop out of 27 patients. Taking the prevalence of diabetic kidney disease as 16.9% margin of error at 5% with confidence interval of 95%, and the power of 80% a minimum sample size of 83 subjects was required to conduct this study.

The diagnosis of diabetes was made as per recommendation of American diabetic association^12^ and diagnosis of diabetic nephropathy was made by performing spot urine protein to check for either the presence of microalbuminuria (30–299 mg/mL) with increased frequency of metabolic syndrome components, stable GFR (creatinine and BUN) and/or macroalbuminuria >300 mg/mL in two of three spot urine specimens, progressive GFR decline, hypertension or any other comorbid.^13^

While patients with chronic systemic disease (Cardiovascular, Urogenital, Immunological etc.), history of DM for >10 years, patients on nephrotoxic drugs, anti-inflammatory drugs and on dialysis were excluded from this study. All procedures were in accordance with the ethical standards of the institutional review committee of Basic Medical Sciences Institute, JPMC Karachi (Ref NO.F.2-81-IRB/2017/GENL/419/JPMC), Pakistan. All participants were informed about the study and gave a written and informed consent to participate. These study participants were divided into the following groups: Group A1 (n=30) diabetes for <5 year without microalbuminuria; Group A2 (n=30) diabetes for 5-10 year with microalbuminuria and Group B (n=25) healthy control subjects. All study participants were followed for 6 months. Blood samples from each participants were obtained in the morning after an overnight fast of 8 hours at baseline and at 6 months follow up. Serum was separated and stored at -80°C and was used to measure complete blood count, blood glucose, HbA1c, serum electrolytes, urea/creatinine levels by automation and KIM-1 levels by sandwich ELISA **(**Glory Science Co., Ltd, Cat. NO 11138). Midstream Urine was collected for dipstick urine albumin test and spot urine albumin at baseline and 6 month follow up.

### Statistical Analysis

Statistical software SPSS version 21.0 was used for data feeding and analysis. A descriptive statistical analysis of continuous variables was performed. Data on continuous variables i.e. biophysical (age, height, weight, BMI, blood pressure, blood glucose etc.) and biochemical (Serum urea, creatinine, electrolytes, and serum KIM-1 etc.) parameters were presented as Mean + standard deviation (SD). One way analysis of variance (ANONA) with post hoc Tukeys test was used to compare groups. Pearson coefficient of correlation (r) was used to relate the levels of serum KIM-1. A p-value ≤0.05 was considered significant.

## RESULTS

Mean age of groups A1 and A2 were comparable (p>0.05) while group B was on the lower age bracket (p=0.01). HbA1c, FBG and RBG levels were higher in diabetic patients as compared to controls (p< 0.005). BUN, Urea creatinine levels were significantly higher in groups A1 and A2 (p<0.01) as compared to controls; whereas no difference was seen for serum electrolytes among groups (p>0.05). The comparison of serum KIM1 levels in study subjects is shown in Table-II. Elevated levels of KIM1 at baseline were seen in patients with more than five year of diabetes as compared to controls and patients with less than five year diabetes (p<0.001). When we compared the levels after 6 months a rising trend was observed for KIM-1 in group A-1 subjects along with the presence of micro albuminuria. This showed that their kidney function was being progressively affected and there was damage to the kidney itself. Moderate positive association were seen for KIM1 with age (r=0.425; p=0.012), creatinine levels (r=0.530; p<0.001), and HbA1c (r=0.576; p<0.001) levels in all patients. While a strong positive association was seen for blood urea nitrogen as a marker for kidney function both at baseline (r= 0.728; p=0.000) and follow up (r=0.747; p=0.001). Multiple logistic regression controlling for age showed that KIM1 was independently associated with BUN (r=0.727; p<0.001), creatinine (r=0.510; p<0.001) and HbA1c (r=0.401; p=0.008) in all groups.

**Table I:** Baseline status of the study subjects.

Variables	A1 Diabetes for <5 year without microalbuminuria (n=30)	A2 Diabetes for 5-10 year with microalbuminuria (n=30)	B Controls (n=25)	p-value
Age (Years)	45.70±10.27^^^	46.72±13.37^#^	29.8±6.4	<0.001
Weight (kg)	69.16±11.44	70.96±16.51	61.86±7.08	0.049
BMI (kg/m^2^)	27.34±5.59^^^	28.21±5.94^#^	22.81±2.69	0.003
Hemoglobin (mg/dL)	12.01±0.98*	10.76±1.87	11.23±0.53	0.005
HbA1c	7.286±0.74^^^	7.43±1.03^#^	5.18±0.27	0.031
FBG at baseline (mg/dl)	136.77 + 45.19*,^^^	172.44 ± 38.32^#^	87.86 ± 9.08	0.031
FBG at 6 months (mg/dl)	172.45 ± 62.56^^^	190.66 ± 71.65^#^	79.55 ± 8.64	0.000
RBG at baseline (mg/dl)	163.77 ± 41.46*	202.71 ± 58.38^#^	156.66 ±58.06	0.007
RBG at 6 months (mg/dl)	150.81 ± 27.35*	233.85 ± 94.86^#^	158.22 ± 32.15	0.005
Blood Urea nitrogen (mg/dL)	11.79±2.75*	25.50±2.12^#^	12.67±3.47	0.000
Serum urea (mg/dL)	35.56±7.19*,^^^	57.30±35.03^#^	27.11±7.42	0.000
Serum Creatinine (mg/dL)	0.73±0.17*	1.34±0.90^#^	0.84± 0.12	0.000
Serum Sodium (mmol/liter)	139.46±2.75	134.26±8.48	132.21±5.44	0.05
Serum Potassium (mmol/liter)	4.36±0.51	3.87±0.84	3.95±0.45	0.05

Values expressed as Mean ± SD. Comparison between groups was made by ANOVA with Tukey’s b applied as a posthoc test analysis.

* A1compared with A2;

^ A1 compared with B;

# A2 compared with B where statistically significant value was set at p<0.05

**Table II:** KIM-1 and urine albumin levels at baseline and follow up.

Variables	A1 (n=30)	A2 (n=30)	B (n=25)	p-value
Baseline KIM -1 Level (ng/ml)	20.91± 14.90 *,^^^	24.86 ± 8.91^#^	7.52 ±0.77	<0.001
Follow up KIM -1 Level (ng/ml)	25.68±14.95^^^	27.34 ± 3.93^#^	8.22 ± 0.83	<0.01
Baseline Urine Albumin (dipstick)	Absent*,^^^	++^#,*^	Absent	0.001
Follow up Urine Albumin (dipstick)	++ ^^^	+++ ^#^	Absent	0.001

Values expressed as Mean ± SD. Comparison between groups was made by ANOVA with Tukey’s b applied as a posthoc test analysis.

* A1compared with A2;

^ A1 compared with B;

# A2 compared with B where statistically significant value was set at p<0.05

## DISCUSSION

This present study aimed at finding out the role of KIM -1, a relatively new marker for diabetic nephropathy. KIM-1 levels progressively rose with the duration of diabetes. This manifestation of elevation of detectable level of serum KIM-1 clearly shows that with the progression of diabetes and increasing duration, there is disruption of renal function and increase in severity of renal inflammation (nephropathy).

Detection of KIM-1 in patients with a shorter duration of diabetes (Group A-1) in this study and elevated levels at six months follow up shows the progression of kidney injury with time. Diabetes is related to the deranged kidney functions that can remain undiagnosed many times. By measuring the levels at two different point in time and with different degree of disease severity in study participants; we were able to show that KIM-1 levels rises with the progression of kidney disease even when all other parameters such as BUN or creatinine level remain within the normal range. This finding is suggestive that as the kidney damage progressed, more KIM-1 is secreted; therefore KIM-1 levels can be used for early detection of DN in diabetic patients.

This is also true in conditions when the micro albuminuria has not been detected in the urine as was the case in this study. Various distinct studies have reported that early renal tubular damage biomarker levels (including urinary KIM-1 levels) are elevated in patients with diabetes, even in those with normal albuminuria.^14^-^16^ This highlights the importance of use of serum KIM-1 from screening perspective of diabetic patient’s for DN during the “tubular phase” of renal damage even before the development of albuminuria. Thus, serum KIM-1 may be recommended as a biomarker for detecting early diabetic nephropathy.^14^,^16^,^17^-^20^ KIM-1 levels in this study showed strong positive association with blood urea nitrogen as a marker for kidney function both at baseline (r= 0.728; p value = 0.000) and follow up (r=0.843; p value = 0.001). Similar results were also observed in a study where they reported dramatic increase in urinary KIM-1 levels within 12 hour of initial ischemic renal insult, prior to the appearance of casts in urine. With this finding it was speculated that KIM-1 is not only a biomarker of kidney injury but also a predictor for the risk of developing ATN.^4^ While moderate positive association were seen for KIM-1 with age (r=0.425; p value = 0.012 and disease duration (r=0.430; p value = 0.011) which is similar to another study.^21^ It has been observed by various researches that longer the duration of diabetes worse is the prevalence of complications of diabetes.^22^,^23^ Serum KIM-1 may become as a highly specific predictor, useful and novel biomarker for early detections of declining renal functions in the diabetes mellitus type 2 patients.

We were however limited to perform renal biopsies to confirm the degree of damage and also were unable to recruit subjects with more than 10 years of disease as most of them suffered from either end stage kidney damage or were on dialysis and were non-compliant to the study follow up.

## CONCLUSION

Rising KIM-1 levels with progressive kidney damage with or without derangement of kidney function is reported in this study. This finding may pave a way towards identifying KIM1 as a prognostic marker for kidney injury.

### Authors’ Contribution

**SSF & FAK:** Designed the study, analyzed the data, wrote the manuscript and approved the final manuscript for submission.

**GMK & SS:** Wrote the manuscript draft and all authors approved the final manuscript for submission.
